# Differences between CEUS LI-RADS and CECT LI-RADS in the diagnosis of focal liver lesions in patients at risk for HCC

**DOI:** 10.1186/s12880-023-01088-1

**Published:** 2023-09-11

**Authors:** Rong Wen, Weiche Huang, Rui Song, Lanhui Qin, Yuquan Wu, Yuting Peng, Xiongyan Huang, Yun He, Hong Yang

**Affiliations:** 1 Department of Medical Ultrasound, The First Affiliated Hospital of Guangxi Medical University, Nanning, Guangxi Zhuang Autonomous Region China; 2 Department of Radiology, The First Affiliated Hospital of Guangxi Medical University, Nanning, Guangxi Zhuang Autonomous Region China; 3 Department of Medical Ultrasound, The Second Affiliated Hospital of Guangxi Medical University, Nanning, Guangxi Zhuang Autonomous Region China; 4 Department of Pathology, The Affiliated Minzu Hospital of Guangxi Medical University, Nanning, Guangxi Zhuang Autonomous Region China

**Keywords:** Hepatocellular carcinoma, Ultrasonography, Computed tomography, Liver Imaging Reporting and Data System

## Abstract

**Objectives:**

To compare the inter-modality consistency and diagnostic performances of the contrast-enhanced ultrasound (CEUS) Liver Imaging Reporting and Data System (LI-RADS) and contrast-enhanced computed tomography (CECT) LI-RADS in patients at risk for hepatocellular carcinoma (HCC), so as to help clinicians to select a more appropriate modality to follow the focal liver lesions (FLLs).

**Methods:**

This retrospective study included untreated 277 FLLs from 247 patients who underwent both CEUS and CECT within 1 month. The ultrasound contrast medium used was SonoVue. FLL categories were independently assigned by two ultrasound physicians and two radiologists using CEUS LI-RADS v2017 and CECT LI-RADS v2018, respectively. The diagnostic performances of CEUS and CECT LI-RADS were evaluated using sensitivity, specificity, positive predictive value (PPV), and negative predictive value. Cohen’s Kappa was employed to evaluate the concordance of the LI-RADS category.

**Results:**

The inter-modality consistency for CEUS and CECT LI-RADS was 0.31 (*p* < 0.001). HCC was more frequently observed in CECT LR-3 and LR-4 hepatic lesions than in CEUS (7.3% vs. 19.5%, *p* < 0.001). The specificity and PPV of CEUS and CECT LR-5 for the diagnosis of HCC were 89.5%, 95.0%, and 82.5%, 94.4%, respectively. The sensitivity of CEUS LR-5 + LR-M for the diagnosis of hepatic malignancies was higher than that of CECT (93.7% vs. 82.7%, *p* < 0.001). The specificity and PPV of CEUS LR-M for the diagnosis of non-HCC malignancies were lower than those of CECT (59.7% vs. 95.5%, *p* < 0.001; 23.4% vs. 70.3%, *p* < 0.001).

**Conclusions:**

The inter-modality consistency between the CEUS and CECT LI-RADS categories is fair. CEUS LI-RADS was more sensitive than CECT LI-RADS in terms of identifying hepatic malignancies, but weaker in terms of separating HCC from non-HCC malignancies.

**Supplementary Information:**

The online version contains supplementary material available at 10.1186/s12880-023-01088-1.

## Background

The early identification of hepatocellular carcinoma (HCC) is essential for improving patient prognosis [1]. Contrast-enhanced ultrasound (CEUS) and contrast-enhanced computed tomography (CECT) both play a critical role in the diagnosis of HCC [2, 3]. The American College of Radiology (ACR) established the CT/MRI Liver Imaging Reporting and Data System (LI-RADS) and the CEUS LI-RADS in 2011 and 2016, respectively, and subsequently updated them to further standardize liver imaging terminology, image acquisition, reporting, and data collection [4]. LI-RADS categorizes focal liver lesions (FLLs) from LR-1 to LR-5 based on their probability of being HCC; clinicians can then give their patients specific recommendations for follow-up or treatment depending on the category [5].

Both CEUS LI-RADS and CT/MRI LI-RADS are used to categorize FLLs in patients who are at a high risk of developing HCC. Even so, it is unknown whether the use of LI-RADS in the interpretation and reporting of FLLs differs depending on which of these two different imaging modalities is used. It is therefore important to compare the inter-modality consistency of LI-RADS from CEUS and CECT as different LI-RADS categories may significantly affect treatments and follow-up plans. Although there have been some previous reports on the concordance of CEUS LI-RADS and CT/MRI LI-RADS, none have compared CEUS LI-RADS with CECT LI-RADS alone to our knowledge [6–9]. It is important to note that, although CT and MRI share a common LI-RADS diagnostic system, the principles of CT and MRI imaging are quite different. Several studies have shown poor concordance between CECT and CEMRI in terms of the primary imaging features and category of LI-RADS [10–12]. As such, mixing CECT and CEMRI together to achieve concordance with CEUS may not actually be representative of the concordance between the CEUS LI-RADS and CECT LI-RADS. The aim of this study was to compare the category concordance and diagnostic performances of the CEUS LI-RADS and CECT LI-RADS in FLLs in order to help clinicians both better understand LI-RADS and select more appropriate examination methods.

## Methods

This study was approved by the Ethics Committee of our hospital; the requirement for individual consent was waived. Patients in our hospital who had undergone both CEUS and CECT liver examinations and had a histopathology of the FLLs from November 2020 to October 2021 were selected (n = 510). A total of 263 patients were excluded for the following reasons: an interval of more than one month between the CEUS and CECT (n = 11), prior treatment for FLLs (n = 82), having FLLs that could not be shown on either US or CECT (n = 9), not being at high risk for HCC as defined by ACR LI-RADS (n = 144), an unclear pathological diagnosis of FLLs (n = 12), and poor image quality (n = 5). Ultimately, 277 FLLs from 247 patients (213 men, 34 women; age range, 26–75 years) were included in this study.

### CEUS protocol

The conventional ultrasound and CEUS examination of the patients’ FLLs was performed using the Mindray RESONA7 (Mindray Bio-Medical Electronics Co., Ltd.) and GE LogiqE9 (GE Healthcare) ultrasound diagnostic instruments. The convex array probe used a frequency range of 1–6 MHz. Harmonic imaging and low mechanical index (MI 0.07–0.10) were applied for CEUS of the liver. The contrast agent (SonoVue; Bracco Suisse SA) was prepared in 5 ml of 0.9% saline and shaken well. The conventional contrast dose used for the liver CEUS was 1.5 ml. A scan of the whole liver was performed prior to starting the CEUS, and any FLLs found were assessed for size, borders, echogenicity, and blood flow, among other factors. Timing started after the antecubital vein bolus of the contrast agent; we recorded dynamic images of CEUS for the first 60 s, and then stored static images with typical enhanced features at 20–30 s intervals. The CEUS examination was dynamically observed for either 3–5 min or until the microbubbles disappeared. If multiple nodules were encountered, repeat contrast injections were required to visualize the remaining FLLs. Each patient’s CEUS images were then exported to mobile hard drives for subsequent analysis.

### CECT protocol

A dual-source CT (SOMATON Definition Flash, Siemens Healthcare) was used to perform both a nonenhanced scan of the patient’s FLLs and scans of the arterial, portal, and late phases. The scanning parameters were as follows: 120 kVp, 400 mAs, layer thickness of 1.5 mm, layer spacing of 1.0 mm. A syringe pump was used to inject the contrast agent (Iohexol, 350 mgI/ml, GE Healthcare) intravenously at a dose of 1.5-2.0 ml/kg of the patient’s body weight and a rate of 3.0-3.5 ml/s. Images of the arterial, portal, and late phases were acquired at 25–30 s, 60–70 s, and 120 s, respectively, after the contrast injection. The CECT images were then stored in the PACS for subsequent analysis.

### Image interpretation

**Fig. 1:**
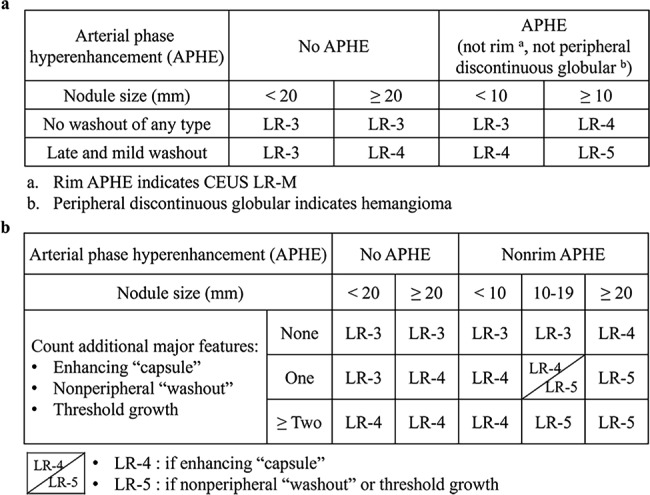
LI-RADS rating criteria. (a) and (b) show the diagnostic tables of CEUS and CECT, respectively. LI-RADS = Liver Imaging Reporting and Data System; CEUS = contrast-enhanced ultrasound; CECT = contrast-enhanced computed tomography

The CEUS images were interpreted by two ultrasound physicians with different levels of experience; Reader 1, W.Y.Q., had over 10 years of liver imaging experience while Reader 2, W.R., had over 3 years of experience at the time of interpretation. The CECT images were interpreted by two radiologists with different levels of experience; Reader 3, S.R., had over 15 years of abdominal CT imaging interpretation experience while Reader 4, Q.L.H., had over 3 years of experience at the time of interpretation. The imaging physicians reviewed the CEUS LI-RADS v2017 and CT/MRI LI-RADS v2018 guidelines prior to interpreting the contrast images. The CEUS and CECT diagnostic tables for LR-3, LR-4, and LR-5 are shown in Fig. 1. The full version can be found on the ACR website (https://www.acr.org/Clinical-Resources/Reporting-and-Data-Systems/LI-RADS). The four physicians assigned the LI-RADS categories independently; although some of the patients’ images had been interpreted by the physicians at the time of the patient’s original visit, the time between the last image interpretation and the current image interpretation was long. Moreover, all of the physicians were unaware of the patients’ clinical information and pathological findings at the time of image interpretation. In clinical practice, if different LI-RADS classifications were obtained for the same FLL in two imaging methods, patients were generally managed or followed up with the higher LI-RADS classification. In order to minimize the effect of inexperience on interpretation, the results of the senior physicians’ interpretations were used to choose between the two imaging methods (Reader 1 vs. Reader 3). We also assessed the concordance of the LI-RADS category between the differently experienced physicians (Reader 1 vs. Reader 2 and Reader 3 vs. Reader 4).

### Statistical analysis

Quantitative data was expressed as the mean ± standard deviation while ranges and qualitative data were expressed as frequencies and percentages. Kappa (κ) and a 95% confidence interval (CI) were used to assess the consistency of the LI-RADS category. Different levels of concordance corresponded to different κ values as follows: 0 to 0.20 - poor concordance; 0.21 to 0.40 - fair concordance; 0.41 to 0.60 - moderate concordance; 0.61 to 0.80 - substantial concordance; 0.81 to 1.00 - almost perfect concordance. Histopathology was defined as the gold standard for diagnosis. The diagnostic performances of the CEUS and CECT LI-RADS were evaluated using sensitivity, specificity, positive predictive value (PPV), and negative predictive value (NPV). The paired chi-square test was used to compare the sensitivity and specificity of the LI-RADS categories between the two imaging methods. The chi-square test was used to compare the PPV and NPV of the LI-RADS categories between the two imaging methods. Propensity score matching was further used to assess the diagnostic performance of CEUS and CECT LI-RADS in identifying benign and malignant hepatic lesion in order to reduce the effect of selection bias. The matched variables were age, gender, and tumor size. Differences were considered statistically significant at *p* < 0.05.

## Results

### Patient characteristics

Table 1 summarizes the clinical characteristics of the 247 patients. Of the 277 lesions included, there were 219 patients with 1 nodule, 26 patients with 2, and 2 patients with 3. An ultrasound-guided biopsy of the hepatic masses was performed in 39 patients while 208 underwent a resection of the hepatic masses. Hepatic malignancies were found in 254 lesions (91.7%); this number was comprised of 220 HCC and 34 non-HCC malignancies. The non-HCC malignancies included 23 intrahepatic cholangiocarcinomas (ICC), 4 combined hepatocellular-cholangiocarcinomas (CHC), 1 angiosarcoma, and 6 metastases (4 derived from digestive tract tumors and 2 derived from cutaneous melanomas). Twenty-three of the masses (8.3%) were benign hepatic lesions.

**Table 1:** Clinical characteristics of the 247 patients

Characteristic	Value
Mean age ± SD (y)*	50.7 ± 10.5 (26–75)
Gender	
Male	213 (86.2)
Female	34 (13.8)
Nodule size	
Mean nodule size ± SD (cm)	5.1 ± 3.5
< 2 cm	43 (15.5)
2.0-4.9 cm	123 (44.4)
≥ 5 cm	111 (40.1)
Number of nodules	
1	219 (88.7)
2	26 (10.5)
3	2 (0.8)
HCC Differentiation (n = 220)	
Well differentiated	49 (22.3)
Moderately differentiated	134 (60.9)
Poorly differentiated	14 (6.4)
NA	23 (10.4)
Liver background	
Chronic hepatitis B viral infection	111 (44.9)
Cirrhosis	136 (55.1)

Note—Unless otherwise indicated, values are numbers of nodules with percentages in parentheses. *: Numbers in parentheses are the range of patient’s age. HCC = hepatocellular carcinoma; NA = not available

### LI-RADS categories and concordance analysis

The comparison of the category-based results of CEUS and CECT LI-RADS is summarized in Table 2. In this study, neither of the two LI-RADS category methods classified hepatic malignancies as either LR-1 or LR-2. For CEUS and CECT LI-RADS, the percentages of hepatic lesions that were categorized as LR-1, LR-3, LR-4, LR-5, and LR-M were 1.8%, 2.9%, 6.1%, 43.0%, and 46.2% vs. 2.5%, 7.6%, 12.3%, 64.3%, and 13.3%, respectively.

**Table 2:** Comparison of the classification results between CEUS and CECT LI-RADS

CECT LI-RADS	CEUS LI-RADS					Total
	1	3	4	5	M	
1	5 (0)	0	0	0	2 (0)	7
3	0	4 (1)	1 (0)	6 (5)	10 (6)	21
4	0	4 (4)	3 (1)	18 (17)	9 (9)	34
5	0	0	12 (9)	91 (90)	75 (69)	178
M	0	0	1 (1)	4 (1)	32 (7)	37
Total	5	8	17	119	128	277

Note—Values are number of hepatic lesions. Values in parentheses are number of HCCs.

**Fig. 2:**
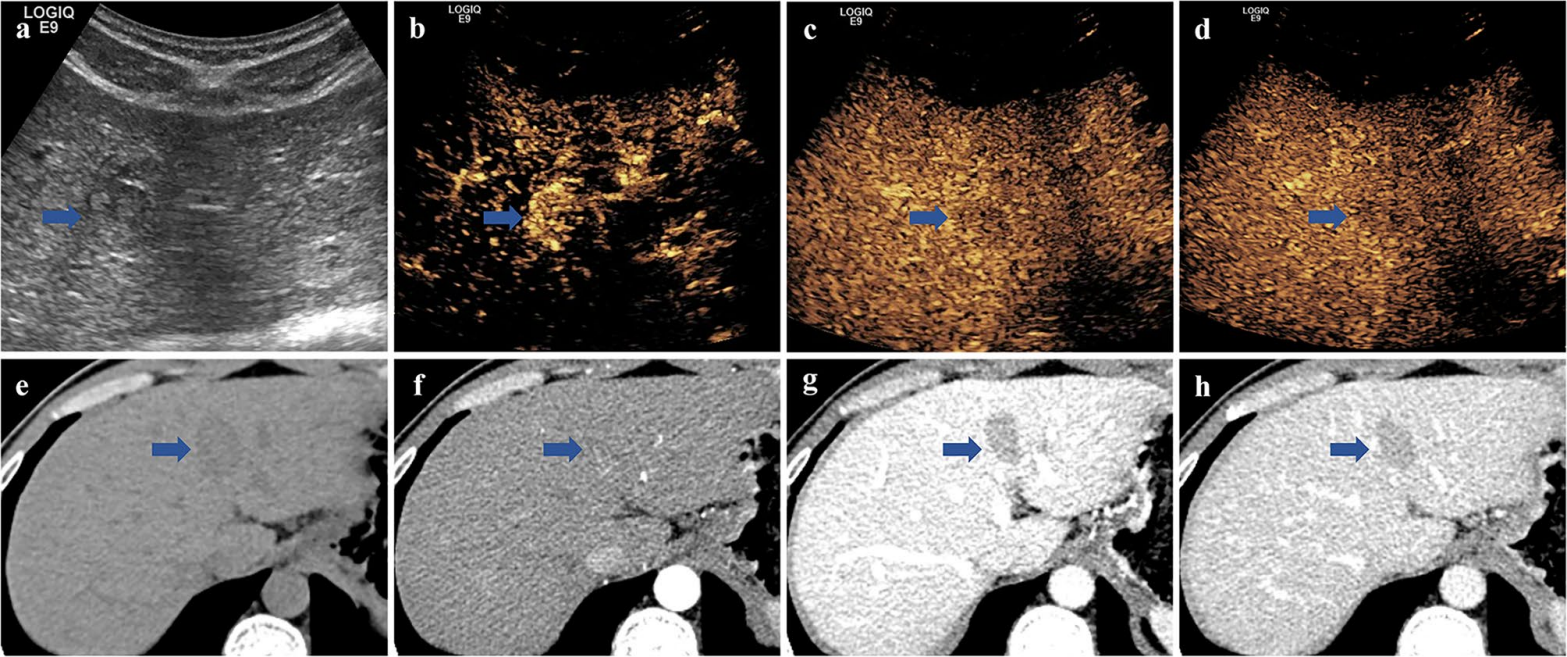
A lesion in segment 4 of the liver that was classified as LR-5 via CEUS and LR-4 via CECT, and eventually pathologically confirmed as HCC in a man with chronic hepatitis B. (a) Conventional ultrasound showed a hypoechoic mass measuring 2.6 × 1.8 cm in segment 4 of the liver. (b) CEUS displayed hyperenhancement in the arterial phase and mild washout in the portal (c) and late (d) phases. (e) Nonenhanced CT displayed a hypodense mass measuring 2.6 × 1.6 cm in segment 4 of the liver. (f) CECT showed isoenhancement in the arterial phase and washout in the portal (g) and late (h) phases

**Fig. 3:**
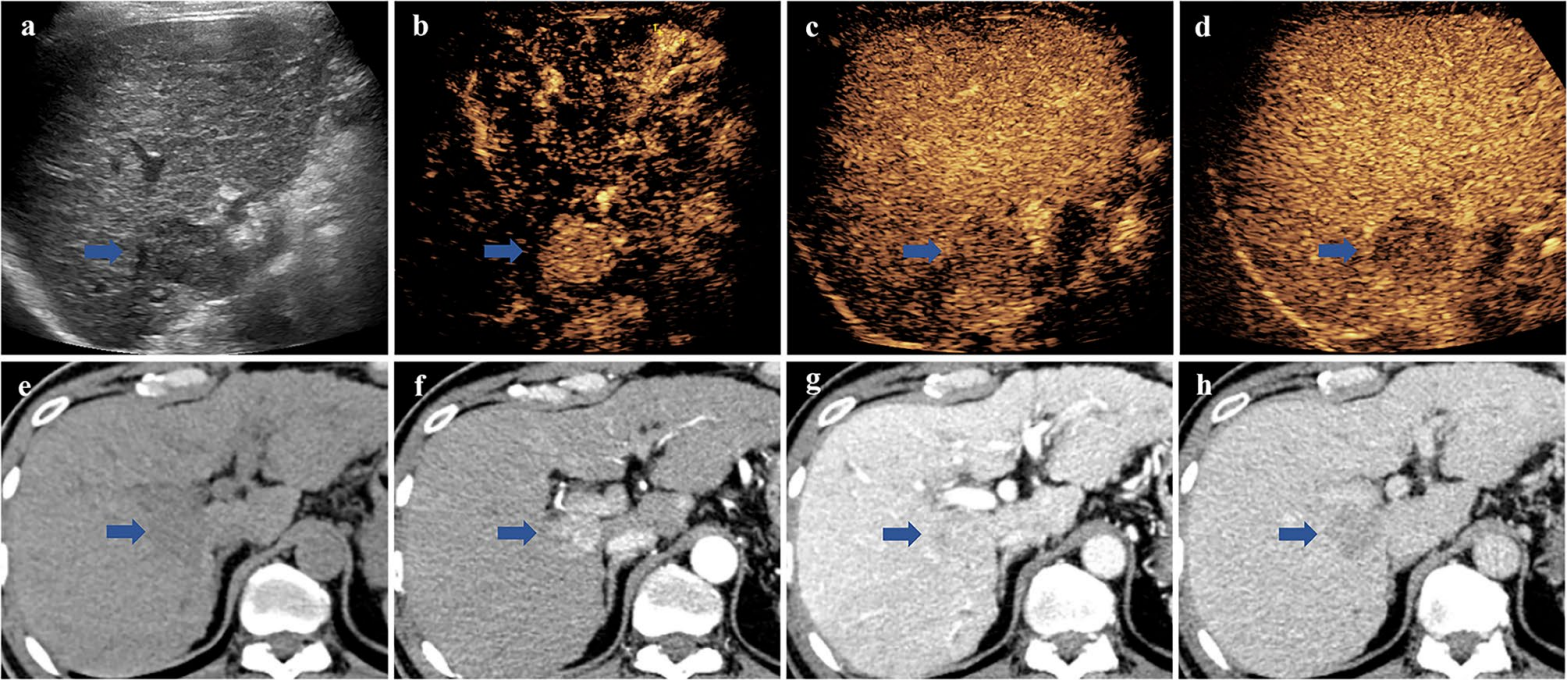
A lesion in segment 5 of the liver that was classified as LR-M via CEUS, LR-5 via CECT, and eventually pathologically confirmed as HCC in a man with hepatitis B-related cirrhosis. (a) Conventional ultrasound showed a hypoechoic mass measuring 3.2 × 2.1 cm in segment 5 of the liver. (b) CEUS displayed hyperenhancement in the arterial phase. (c) CEUS displayed early washout (42 s after injection) in the portal phase and (d) mild washout in the late phase. (e) Nonenhanced CT showed a hypodense mass measuring 2.9 × 2.8 cm in segment 5 of the liver. (f) CECT showed hyperenhancement in the arterial and portal (g) phases and washout in the late (h) phase with a visible enhancing capsule

For CEUS LI-RADS, 7.3% (16/220) of the HCCs were categorized as either LR-3 or LR-4, 51.4% (113/220) as LR-5, and 41.3% (91/220) as LR-M. Three ICC and one metastasis were also classified as LR-5. For CECT LI-RADS, 19.5% (43/220) of the HCCs were categorized as either LR-3 or LR-4, 76.4% (168/220) as LR-5, and 4.1% (9/220) as LR-M. Four ICC, two CHC, and one metastasis were also categorized as LR-5. Twenty-two HCCs were classified as either LR-3 or LR-4 by CECT and LR-5 by CEUS; the average nodule size of these HCCs was 2.8 cm. Nine HCCs were classified as either LR-3 or LR-4 by CEUS and LR-5 by CECT; the average nodule size of these HCCs was 2.3 cm. Examples of the LI-RADS classification of FLLs in CEUS and CECT are shown in Figs. 2 and 3.

The inter-observer consistency for CEUS LI-RADS and CECT LI-RADS were 0.74 (95% CI: 0.66, 0.81, *p* < 0.001) and 0.80 (95% CI: 0.72, 0.88, *p* < 0.001), respectively. The inter-modality consistency for CEUS LI-RADS and CECT LI-RADS was 0.31 (95% CI: 0.21, 0.42, *p* < 0.001). Table 3 summarizes the pathological results of the 277 hepatic nodules and their distribution across the LI-RADS classification.

**Table 3:** Pathological results for the 277 hepatic nodules and their distribution in the LI-RADS category

Pathological results	CEUS LI-RADS (CECT LI-RADS)					Total
	1	3	4	5	M	
HCC	0 (0)	5 (12)	11 (31)	113 (168)	91 (9)	220
ICC	0 (0)	0 (1)	0 (0)	3 (4)	20 (18)	23
CHC	0 (0)	0 (0)	0 (0)	0 (2)	4 (2)	4
Angiosarcoma	0 (0)	0 (0)	0 (0)	0 (0)	1 (1)	1
Metastasis	0 (0)	0 (0)	0 (0)	1 (1)	5 (5)	6
Dysplastic nodule	0 (0)	0 (1)	4 (2)	0 (1)	0 (0)	4
Regenerative nodule	0 (0)	0 (0)	1 (0)	0 (1)	0 (0)	1
Hemangioma	5 (7)	1 (1)	0 (0)	0 (0)	2 (0)	8
FNH	0 (0)	0 (0)	0 (1)	1 (0)	0 (0)	1
Adenoma	0 (0)	0 (1)	1 (0)	0 (1)	1 (0)	2
Angiomyolipoma	0 (0)	0 (1)	0 (0)	1 (0)	0 (0)	1
Steatosis	0 (0)	1 (1)	0 (0)	0 (0)	0 (0)	1
GSD	0 (0)	0 (1)	0 (0)	0 (0)	1 (0)	1
Necrotic nodule	0 (0)	1 (1)	0 (0)	0 (0)	1 (1)	2
Liver abscess	0 (0)	0 (1)	0 (0)	0 (0)	1 (0)	1
MCN	0 (0)	0 (0)	0 (0)	0 (0)	1 (1)	1
Total	5 (7)	8 (21)	17 (34)	119 (178)	128 (37)	277

Note—Values are number of hepatic lesions. HCC = hepatocellular carcinoma; ICC = intrahepatic cholangiocarcinoma; CHC = combined hepatocellular-cholangiocarcinoma; FNH = focal nodular hyperplasia; GSD = glycogen storage disease; MCN = mucinous cystic neoplasm

#### Diagnostic accuracy of CEUS and CECT LI-RADS

The diagnostic performances of the CEUS and CECT LI-RADS for HCC and non-HCC malignancies are summarized in Table 4. The specificity and PPV of CEUS LR-5 and CECT LR-5 for the diagnosis of HCCs were 89.5% (95% CI: 78.5%, 96.0%), 95.0% (95% CI: 89.7%, 97.6%) vs. 82.5% (95% CI: 70.1%, 91.3%), 94.4% (95% CI: 90.5%, 96.7%).

**Table 4:** Diagnostic performances of CEUS and CECT LI-RADS for HCC and non-HCC malignancies

Variables	HCC			Non-HCC malignancies		
	CEUS LR-5	CECT LR-5	*p*	CEUS LR-M	CECT LR-M	*p*
TP	113	168		30	26	
TN	51	47		145	232	
FP	6	10		98	11	
FN	107	52		4	8	
Sens. (%)	51.4 (44.6, 58.1)	76.4 (70.2, 81.8)	< 0.001	88.2 (72.6, 96.7)	76.5 (58.8, 89.3)	0.344
Spec. (%)	89.5 (78.5, 96.0)	82.5 (70.1, 91.3)	0.424	59.7 (53.2, 65.9)	95.5 (92.1, 97.7)	< 0.001
PPV (%)	95.0 (89.7, 97.6)	94.4 (90.5, 96.7)	0.830	23.4 (20.1, 27.1)	70.3 (56.3, 81.3)	< 0.001
NPV (%)	32.3 (28.8, 35.9)	47.5 (40.9, 54.1)	0.015	97.3 (93.5, 98.9)	96.7 (94.1, 98.2)	0.719

Note—Values in parentheses are 95% confidence intervals. TP = true positive; TN = true negative; FP = false positive, FN = false negative; Sens. = Sensitivity; Spec. = specificity

The specificity and PPV of CEUS LR-M and CECT LR-M for the diagnosis of non-HCC malignancies were 59.7% (95% CI: 53.2%, 65.9%), 23.4% (95% CI: 20.1%, 27.1%) vs. 95.5% (95% CI: 92.1%, 97.7%), 70.3% (95% CI: 56.3%, 81.3%). The diagnostic performances of CEUS and CECT LI-RADS for HCC and non-HCC malignancies in ≤ 5 cm FLLs are also shown in Supplemental Table 1.

The diagnostic performances of the CEUS and CECT LI-RADS for hepatic malignancies are summarized in Table 5. The sensitivity and specificity of the CEUS LR-5 + LR-M and CECT LR-5 + LR-M for the diagnosis of hepatic malignancies were 93.7% (95% CI: 90.0%, 96.4%), 60.9% (95% CI: 38.5%, 80.3%) vs. 82.7% (95% CI: 77.5%, 87.1%), 78.3% (95% CI: 56.3%, 92.5%). After one-to-two (benign: malignant = 1:2) propensity score matching, the diagnostic performances of the CEUS and CECT LI-RADS for hepatic malignancies are shown in Supplemental Table 2.

**Table 5:** Diagnostic performances of CEUS and CECT LI-RADS for hepatic malignancies

Variables	CEUS LR-5 + LR-M	CECT LR-5 + LR-M	*p*
TP	238	210	
TN	14	18	
FP	9	5	
FN	16	44	
Sensitivity (%)	93.7 (90.0, 96.4)	82.7 (77.5, 87.1)	< 0.001
Specificity (%)	60.9 (38.5, 80.3)	78.3 (56.3, 92.5)	0.344
PPV (%)	96.4 (94.1, 97.8)	97.7 (95.1, 98.9)	0.410
NPV (%)	46.7 (33.0, 60.9)	29.0 (22.5, 36.6)	0.098

Note—Values in parentheses are 95% confidence intervals. TP = true positive; TN = true negative; FP = false positive, FN = false negative

## Discussion

In this study, we compared the concordance and diagnostic performance of CEUS LI-RADS and CECT LI-RADS by analyzing 277 untreated FLLs in patients at risk for HCC. Our results indicated that both CEUS and CECT LR-5 perform well when it comes to diagnosing HCC. However, the fair concordance between the CEUS and CECT LI-RADS categories suggests that the same LI-RADS classifications cannot be assumed to fully equivalent; further analysis of their differences is needed. A meta-analysis of 11 studies that consisted of 5535 FLLs with 3983 HCCs showed that the specificity and PPV of CEUS LR-5 for diagnosing HCC were 92% and 97%, respectively [13]. Another meta-analysis of 14 studies that consisted of 2708 FLLs with 1841 HCCs showed that the specificity and PPV of CECT LR-5 for diagnosing HCC were 91% and 95%, respectively [14]. Our results showed that both CEUS and CECT LR-5 showed high specificity (89.5% vs. 82.5%) and PPV (95.0% vs. 94.4%) in the diagnosis of HCC; these results are generally line with previous studies, with the exception of the fact that the specificity of CECT LR-5 was a bit lower than usually seen in the literature [14]. Additionally, the inter-observer consistency in our comparison categories was substantial for both CEUS and CECT (κ, 0.74 and 0.81) for the LI-RADS. These results indicate a strong diagnostic concordance between practitioners with different levels of experience for both CEUS LI-RADS and CECT LI-RADS, suggesting that these diagnostic algorithms can be applied by different observers with reproducible consistency.

In analyzing the category concordance of the two modalities, our results showed a fair concordance between CEUS LI-RADS and CECT LI-RADS; this is in line with previous findings [6, 7]. In our study, HCC was more frequently observed in CECT LR-3 and LR-4 hepatic lesions than in CEUS (7.3% vs. 19.5%, *p* < 0.001), and was primarily concentrated in small hepatic nodules. We found that arterial phase hyperenhancement and late and mild washout, all of which are important imaging features assigned to LR-5, were more frequently observed with CEUS than with CECT, suggesting that CEUS is more sensitive to these factors in FLLs than CECT is. CECT only scans at fixed time points, which could lead to potentially missing some key imaging features, while CEUS displays the early arterial enhancement features of FLLs in real time, essentially eliminating the possibility of misdiagnosis due to the time interval between CT scans in the arterial phase [15]. In addition, the CT contrast agent spreads into the tumor interstitium in either the portal or late phase and may obscure the washout of hepatic masses in some FLLs; conversely, CEUS uses a pure blood pool contrast agent that appears as a true washout in the hepatic masses, making it easier to detect signs of washout [16]. This suggests that the enhancement pattern of FLLs can be detected by CEUS for CECT LR-3 and LR-4 nodules in cases of subsequent investigations or follow-ups, allowing for the development of a more appropriate treatment and follow-up plan for the patient.

Identifying either the benign or malignant nature of FLLs is one of the important jobs of imaging. Although CEUS has significantly improved the diagnostic performance of conventional ultrasound in this regard, CT and MRI still dominate the field because CEUS has not been developed for a long time. This study showed that CEUS LI-RADS was more sensitive than CECT LI-RADS when CEUS and CECT LR-5 + LR-M were used as criteria for the diagnosis of hepatic malignancies, though differences in specificity, PPV, and NPV were not statistically significant. After further balancing the number of benign and malignant hepatic nodules, the assessment of diagnostic performance was performed again and similar results were obtained. This suggests that CEUS LI-RADS may be superior to CECT LI-RADS in terms of identifying the manifestations of hepatic malignancies. However, in distinguishing HCC from non-HCC malignancies, our study found that the diagnostic performance of CEUS LI-RADS was weaker than that of CECT LI-RADS. The specificity and PPV of CEUS LR-M for non-HCC malignancies was only 59.7% and 23.4%, as compared to 95.5% and 70.3% for CECT LR-M; both results were statistically significant. CEUS LR-M, at minimum, requires one of the subsequent three contrast features: rim arterial phase hyperenhancement, early washout (< 60 s), and marked washout (< 120 s). We believe that it is primarily HCC (71.1%, 91/128) that is overrepresented in LR-M. Upon further analysis, we found that 82.4% (75/91) of HCCs were categorized as CEUS LR-M because of early washout alone; this is in line with previous studies [17, 18]. Li et al. [19] revealed that the specificity of CEUS LR-M could be further improved without reducing its sensitivity if the start time for early washout was adjusted to < 45 s while Zheng et al. [18] proposed a reclassification of LR-5 for liver nodules categorized as CEUS LR-M due to early washout, provided that there was no punched-out appearance within 5 min, would improve diagnostic performance in terms of differentiating HCC and non-HCC malignancies. This suggests that the present standard for the CEUS LR-M should be further refined, and the efficiency of HCC diagnoses could be improved via the reduction of CEUS LR-M misclassifications [20]. Therefore, FLLs with the current standard CEUS LR-M could be further assessed using CECT examination.

This study had some limitations. First, our study was retrospective, which can lead to selection bias. Future prospective and multicenter studies are therefore necessary to validate these results. Second, this study used pathology as the gold standard, resulting in the inclusion of fewer benign hepatic lesions. In addition, if the diagnosis of one contrast-enhanced examination tends to be benign, it is generally unlikely that another contrast-enhanced examination will be performed at the same time, which will further reduce the number of benign hepatic lesions. Third, due to the operator-dependent nature of ultrasound, the CEUS LI-RADS v2017 makes it clear that it is currently only applicable to liver lesions that can be visualized by conventional ultrasound. The exclusion of liver lesions not visible on ultrasound or CT in this study will inevitably have some impact on the results, but we believe that the small number of patients excluded because of the invisibility of one of the modalities (US = 6, CT = 3) will not affect the overall results.

## Conclusions

In conclusion, both CEUS LI-RADS and CECT LI-RADS showed strong diagnostic performances for HCC and were effective diagnostic tools for detecting HCC. In addition, the inter-modality consistency between the CEUS LI-RADS and CECT LI-RADS categories was fair. For other LI-RADS categories, such as CECT LR-3, LR-4, or CEUS LR-M, the selection of appropriate imaging examinations will help improve the diagnosis of HCC and non-HCC malignancies.

### Electronic supplementary material

Below is the link to the electronic supplementary material.

Supplementary Material 1

## Data Availability

The dataset and analysis for this study can be obtained from the corresponding author upon reasonable request.

## Abbreviations

HCC: Hepatocellular carcinoma
CEUS: Contrast-enhanced ultrasound
CECT: Contrast-enhanced computed tomography
ACR: American College of Radiology
LI-RADS: Liver Imaging Reporting and Data System
FLLs: Focal liver lesions
CI: Confidence interval
κ: Kappa
PPV: Positive predictive value
NPV: Negative predictive value
ICC: Intrahepatic cholangiocarcinoma
CHC: Combined hepatocellular-cholangiocarcinoma
