# Supplementary material for: Differences between CEUS LI-RADS and CECT LI-RADS in the diagnosis of focal liver lesions in patients at risk for HCC
Source: BMC Med Imaging. 2023 Sep 11;23:122. doi: 10.1186/s12880-023-01088-1 (PMC10496202; doi:10.1186/s12880-023-01088-1)
Supplement: Supplementary file 1 — Supplementary Material 1 [file 12880_2023_1088_MOESM1_ESM.docx]

**Supplemental Table 1** Diagnostic performances of CEUS and CECT LI-RADS for HCC and non-HCC malignancies in ≤ 5 cm FLLs

| Variables | HCC | |  | Non-HCC malignancies | |  |
| --- | --- | --- | --- | --- | --- | --- |
|  | CEUS LR-5 | CECT LR-5 | *p* | CEUS LR-M | CECT LR-M | *p* |
| TP | 75 | 96 |  | 14 | 13 |  |
| TN | 28 | 26 |  | 101 | 149 |  |
| FP | 4 | 6 |  | 51 | 3 |  |
| FN | 62 | 41 |  | 3 | 4 |  |
| Sens. (%) | 54.7 (46.0, 63.3) | 70.1 (61.7, 77.6) | 0.010 | 82.4 (56.6, 96.2) | 76.5 (50.1, 93.2) | 1.000 |
| Spec. (%) | 87.5 (71.0, 96.5) | 81.3 (63.6, 92.8) | 0.754 | 66.4 (58.3, 73.9) | 98.0 (94.3, 99.6) | <0.001 |
| PPV (%) | 94.9 (88.1, 97.9) | 94.1 (88.5, 97.1) | 0.812 | 21.5 (16.7, 27.3) | 81.3 (57.8, 93.2) | <0.001 |
| NPV (%) | 31.1 (26.5, 36.1) | 38.8 (31.8, 46.3) | 0.317 | 97.1 (92.3, 99.0) | 97.4 (94.1, 98.9) | 0.869 |

Note—Values in parentheses are 95% confidence intervals. FLLs = focal liver lesions; TP = true positive; TN = true negative; FP = false positive, FN = false negative; Sens. = Sensitivity; Spec. = Specificity.

**Supplemental Table 2** Diagnostic performances of CEUS and CECT LI-RADS for hepatic malignancies after propensity score matching

| Variables | CEUS LR-5+LR-M | CECT LR-5+LR-M | *p* |
| --- | --- | --- | --- |
| TP | 42 | 34 |  |
| TN | 14 | 18 |  |
| FP | 9 | 5 |  |
| FN | 4 | 12 |  |
| Sensitivity (%) | 91.3 (79.2, 97.6) | 73.9 (58.9, 85.7) | 0.039 |
| Specificity (%) | 60.9 (38.5, 80.3) | 78.3 (56.3, 92.5) | 0.344 |
| PPV (%) | 82.4 (73.6, 88.7) | 87.2 (75.4, 93.8) | 0.534 |
| NPV (%) | 77.8 (56.5, 90.4) | 60.0 (46.8, 71.9) | 0.178 |

Note—Values in parentheses are 95% confidence intervals. TP = true positive; TN = true negative; FP = false positive, FN = false negative.
